# Skin-Associated Secondary Plasmacytomas in Multiple Myeloma: A Rare Entity With Prognostic Relevance

**DOI:** 10.7759/cureus.101330

**Published:** 2026-01-12

**Authors:** Rihame Alheyasat, Zakia Douhi, Aimane Zaim, Layla Tahiri El Ousrouti, Fatima Zahra Mernissi

**Affiliations:** 1 Dermatology, Hassan II University Hospital, Sidi Mohamed Ben Abdellah University, Fez, MAR; 2 Pathology, Hassan II University Hospital, Sidi Mohamed Ben Abdellah University, Fez, MAR

**Keywords:** extramedullary plasmacytoma, metastatic skin lesions, multiple myeloma, prognosis, secondary cutaneous plasmacytoma

## Abstract

Extramedullary plasmacytomas rarely involve the skin, and secondary cutaneous plasmacytomas (SCPs) are typically observed in advanced multiple myeloma (MM). We report a case of a 58-year-old man with known MM who developed multiple violaceous nodules and right-sided exophthalmos. Skin biopsy showed a kappa-restricted plasmacytic infiltrate, and imaging revealed extensive soft-tissue masses, widespread osteolytic lesions, and an intraorbital tumor. These findings established a diagnosis of metastatic SCP associated with progressive MM. Despite initiation of VDT-PACE chemotherapy (bortezomib, dexamethasone, thalidomide, cisplatin, doxorubicin, cyclophosphamide, and etoposide), the patient’s condition rapidly deteriorated, leading to death after four cycles. This case highlights the rarity and severity of SCP and underscores the importance of recognizing cutaneous involvement as a marker of aggressive, treatment-refractory MM and imminent systemic progression.

## Introduction

According to the International Myeloma Working Group (IMWG), plasmacytoma is a rare, discrete tumor of monoclonal plasma cells that may present either as a solitary bone lesion (solitary bone plasmacytoma) or as an extramedullary mass in the absence of systemic disease. This entity is typically characterized by a normal or near-normal bone marrow containing less than 5% of plasma cells, absence of myeloma-defining CRAB features (hypercalcemia, renal failure, anemia, and osteolytic bone lesions), and a small or absent serum or urine monoclonal paraprotein [1]. The diagnosis of solitary plasmacytoma requires strict adherence to IMWG criteria, including a biopsy-confirmed solitary lesion with clonal plasma cells, normal bone marrow without evidence of clonal plasma cells, normal skeletal imaging except for the primary lesion, and absence of end-organ damage [1].

Among extramedullary plasmacytomas (EMPs), secondary cutaneous plasmacytomas (SCP) represent skin lesions specifically associated with multiple myeloma (MM). First described by Hedinger in 1911 [2], these lesions may develop via lymphatic or vascular dissemination or, more commonly, by direct extension from underlying bone lesions. They reflect a clonal proliferation of extramedullary plasma cells producing monoclonal immunoglobulins.

Cutaneous plasmacytomas (CPs) are exceptionally rare [3-5]. SCPs are distinguished from primary CPs by their association with MM and are most frequently observed in advanced stages of the disease, generally carrying a poor prognosis. Accurate differentiation from cutaneous lymphomas is essential due to differences in diagnosis, treatment, and outcomes [6].

In some cases, SCP may represent the initial manifestation of MM [7], highlighting the need for early recognition of these rare dermatologic presentations. Their occurrence generally indicates aggressive disease and poor outcomes, with survival often limited to less than 12 months after diagnosis [8].

We report a case of SCP in a patient with known MM, revealing systemic tumor progression and a fatal outcome.

## Case presentation

A 58-year-old man with a history of IgG-type MM was initially treated with six cycles of the VTd regimen (Velcade^®^ (bortezomib), thalidomide, and dexamethasone) combined with zoledronic acid and subsequently received thalidomide maintenance therapy. He presented to the emergency department with right-sided exophthalmos and, over the preceding four months, multiple nodular cutaneous lesions on the thorax, accompanied by progressive weight loss of approximately eight kilograms.

**Figure 1:**
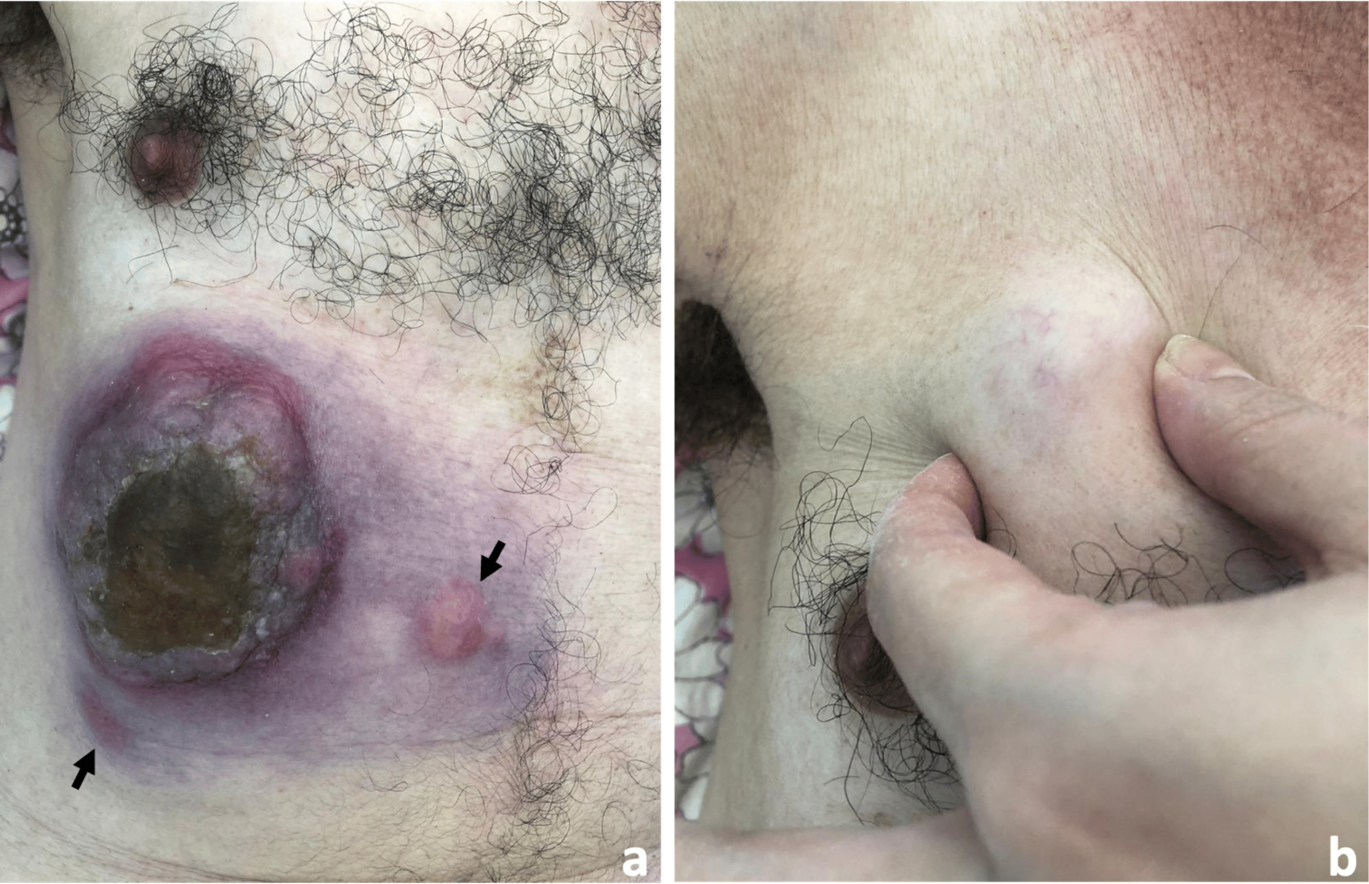
(a) Multilobulated erythematous–violaceous tumor located in the right hypochondriac region, exhibiting partial surface erosion and honey-colored crusts, and surrounded by multiple adjacent erythematous nodules indicated by black arrows. (b) Adjacent peri-tumoral subcutaneous nodule

On general examination, the patient appeared clinically deteriorated but remained afebrile. Dermatologic assessment revealed, in the right hypochondriac region, a 15-cm erythematous-violaceous tumor with a multilobulated surface, firm consistency, and fixation to underlying tissues. The lesion was partially eroded and covered by honey-colored crusts. The surrounding skin showed erythematous-bluish discoloration with palpable induration (Figure 1a). Several adjacent subcutaneous nodules were also noted, particularly within major skin folds and across the back and thoracic region (Figure 1b).

**Figure 2:**
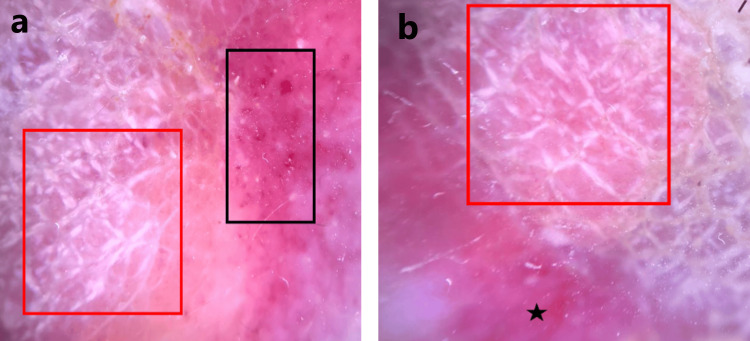
Dermoscopy revealed homogeneous violaceous-red structureless areas (black star, b) intersected by shiny white streaks (red squares, a and b) and globular vessels (black square, a).

Dermoscopy demonstrated homogeneous violaceous-red structureless areas, intersecting shiny white streaks, and globular vessels (Figure 2).

**Figure 3:**
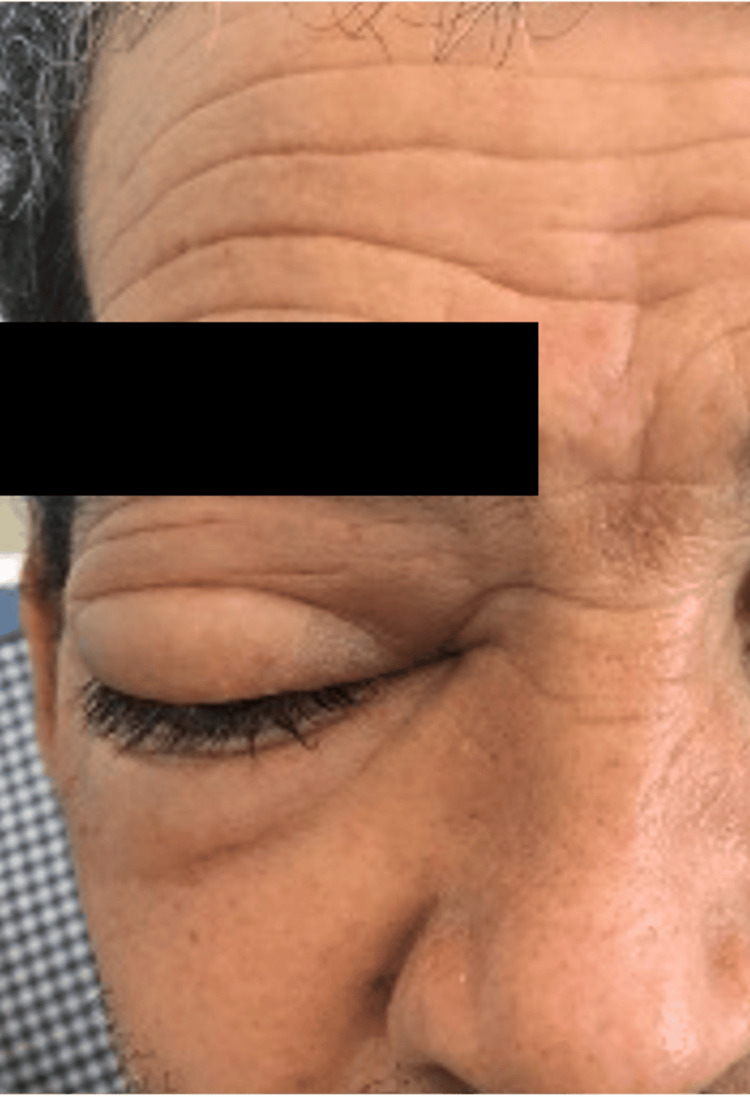
Prominent tumefaction of the right eyelid

Ophthalmologic examination confirmed an irreducible, non-pulsatile right exophthalmos without signs of inflammation (Figure 3).

The initial laboratory investigations are summarized in Table 1.

**Table 1:** Relevant initial laboratory findings CRAB, hypercalcemia, renal failure, anemia, and osteolytic bone lesions.

Parameter	Result	Reference range
Serum protein electrophoresis		
Albumin	40 g/L	35–50 g/L
Alpha-1 globulin	3.9 g/L	2–5 g/L
Alpha-2 globulin	6.8 g/L	5–9 g/L
Beta-1 globulin	4.1 g/L	3–5 g/L
Beta-2 globulin	2.8 g/L	1.5–3 g/L
Gamma globulin	15.3 g/L (M protein: 11.4)	7–13 g/L
Immunoglobulins		
Immunoglobulin G	18 g/L	7–16 g/L
Immunoglobulin A	1.17 g/L	0.7–4 g/L
Immunoglobulin M	0.27 g/L	0.4–2.3 g/L
Complete blood count—CRAB criteria		
Hemoglobin	10 g/dL	12–16 g/dL
Mean corpuscular volume	82 fL	80–100 fL
Mean corpuscular hemoglobin concentration	34 g/dL	32–36 g/dL
White blood cells	3,060/µL	4,000–10,000/µL
Neutrophils (polymorphonuclear neutrophils)	1,420/µL	1,500–7,500/µL
Lymphocytes	1,000/µL	1,000–4,800/µL
Platelets	225,000/µL	150,000–400,000/µL
Biochemistry—CRAB criteria		
Serum calcium	8.3 mg/dL	8.5–10.5 mg/dL
Serum albumin	36 g/L	35–50 g/L
Serum creatinine	0.7 mg/dL	0.6–1.3 mg/dL
Serum urea	0.35 g/L	0.2–0.5 g/L

**Figure 4:**
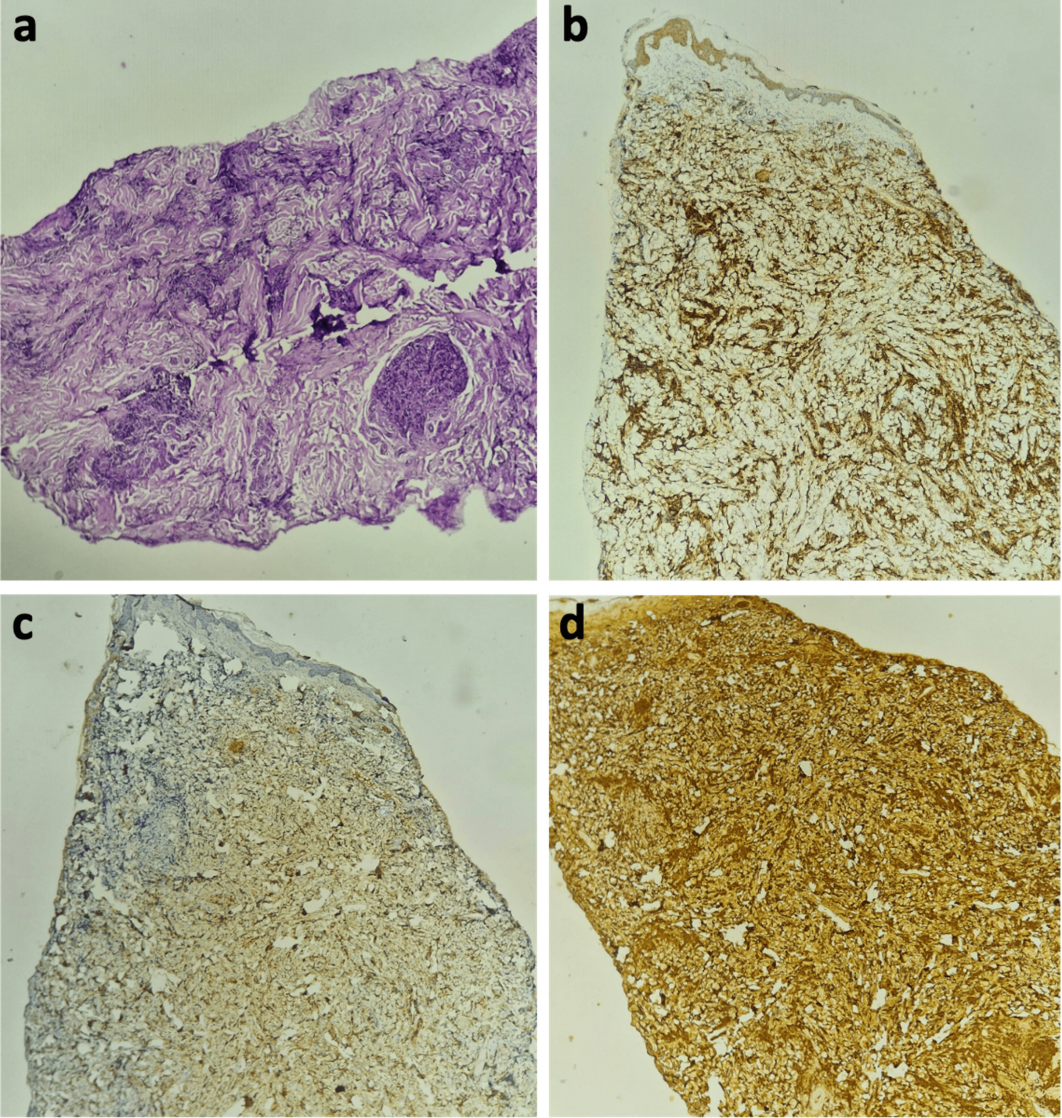
(a) H&E stain at ×200 magnification showing a dense dermal infiltrate of atypical plasma cells with round, hyperchromatic nuclei and abundant cytoplasm. (b) Immunohistochemical staining demonstrating intense and diffuse expression of CD138 by plasma cells. (c) Immunohistochemical staining revealing weak and focal expression of lambda light chains. (d) Immunohistochemical staining showing intense and diffuse expression of kappa light chains. CD, cluster of differentiation.

Histopathologic examination of a skin biopsy revealed a dense dermal infiltrate composed of sheets of atypical plasmacytoid cells with round to oval hyperchromatic nuclei and abundant eosinophilic cytoplasm (Figure 4a). Immunohistochemistry demonstrated strong and diffuse expression of cluster of differentiation (CD)138 (Figure 4b), minimal expression of lambda light chain (Figure 4c), and strong, diffuse expression of kappa light chain (Figure 4d). CD20 and CD3 highlighted reactive B and T lymphocytes. These findings were consistent with a kappa-restricted CP.

**Figure 5:**
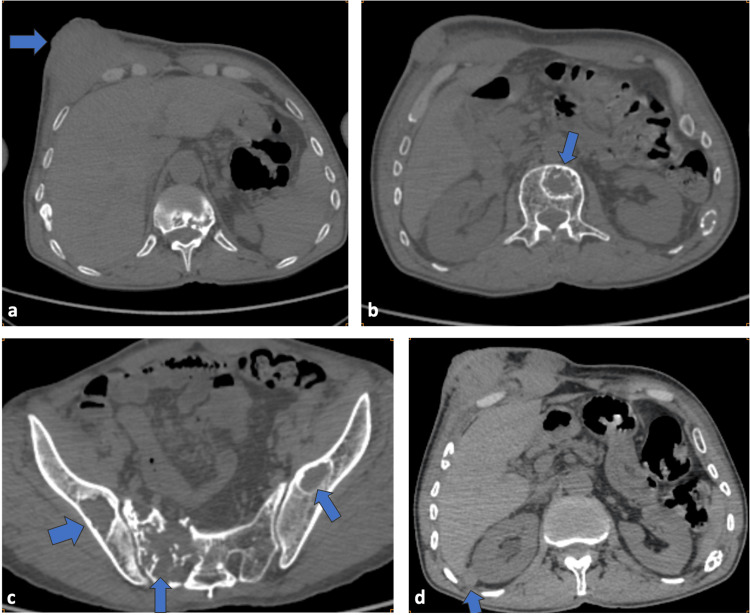
CT scan imaging showing: (a) Large anterior thoracoabdominal parietal soft-tissue tumor (blue arrow). (b) Osteolytic metastatic lesion involving the vertebral body (blue arrow). (c) Multiple osteolytic metastatic lesions involving the pelvic girdle (blue arrows). (d) Right adrenal metastatic nodule in the lateral limb, measuring 20 × 12 mm (blue arrow)

Radiologic evaluation revealed multiple tumors affecting the thoracoabdominal walls (Figure 5a) and abdominal cavity, as well as numerous osteolytic lesions affecting nearly the entire axial skeleton (Figure 5b) and pelvic girdle (Figure 5c). Additional secondary lesions were identified in the peritoneum, adrenal glands (Figure 5d), and thoracic and abdominal walls, along with a right intraorbital soft-tissue mass responsible for grade III exophthalmos. A small volume of intraperitoneal effusion was also present.

A diagnosis of metastatic CP associated with progressive MM was established. Due to clinical and radiologic progression, chemotherapy was reinitiated using the VDT-PACE regimen (bortezomib, dexamethasone, thalidomide, cisplatin, doxorubicin, cyclophosphamide, and etoposide) combined with pulse corticosteroid therapy.

Despite aggressive treatment, the patient died after four cycles of chemotherapy.

## Discussion

Plasmacytomas are neoplastic proliferations of monoclonal plasma cells. They are relatively uncommon, accounting for 1%-2% of plasma cell dyscrasias, and may involve either bone or soft tissues, corresponding respectively to solitary bone plasmacytoma or EMP [9]. EMPs are associated with MM in approximately 70% of autopsy cases [3]. CPs, however, remain exceptionally rare. Secondary cutaneous EMP, which is more frequent than primary forms, generally arises late in the course of MM. They most often develop in proximity to underlying osteolytic lesions and less commonly at distant cutaneous sites [10,11]. When multiple lesions are present, they are classified as metastatic SCPs (MSCPs).

According to Alberts and Lynch, the mean age of patients with secondary EMP is similar to that of patients with MM and without cutaneous involvement [12]. Clinically, CP typically presents as non-pruritic dermal papules or nodules measuring 1-5 cm, with red to violaceous coloration that may appear ecchymotic. Peripheral inflammation or ulceration may be present, as in our patient. Distinguishing primary from secondary lesions is challenging based solely on clinical presentation, although secondary forms tend to be more aggressive, nodular, or infiltrative [7]. Lesions may develop on any cutaneous site, with 49.5% located on the trunk and 20% on the head and neck region [8,13].

Histopathologically, CP generally show a dense dermal infiltrate of mature, monomorphic plasma cells with minimal cytologic atypia, often extending into the subcutis while sparing the superficial dermis and epidermis. Immunohistochemistry typically demonstrates strong expression of CD138, CD79a, and CD38, with a lack of CD20 and CD19 expression. Assessment of light-chain restriction (kappa or lambda) is essential for confirming monoclonality [14,15]. Histopathology alone cannot reliably distinguish primary from secondary CP; however, the presence of amyloid deposition generally favors a secondary origin.

MSCP usually occurs in patients with advanced, high-tumor burden MM, as illustrated in our case, and it is associated with rapidly progressive and often fatal disease. In the series by Alberts and Lynch, 50% of patients died within six months and 75% within two years [15]. Our patient similarly experienced a rapid clinical decline, with death occurring six months after the onset of cutaneous dissemination. To date, no standardized therapeutic strategy exists for MSCP. Nevertheless, systemic chemotherapy, often combined with radiotherapy for localized symptomatic lesions, remains the most commonly employed approach, although outcomes remain poor.

## Conclusions

Cutaneous involvement in MM, particularly in the form of MSCP, appears to be associated with aggressive disease biology and poor prognosis. Our case reinforces the possibility that CP may represent a clinical marker of advanced, treatment-refractory MM and could potentially signal imminent systemic progression. Whether cutaneous dissemination constitutes an independent prognostic factor or simply reflects a high tumor burden remains uncertain. Larger multicenter studies and prospective analyses are needed to clarify its prognostic value, identify high-risk patient subsets, and guide the development of more effective therapeutic strategies for this rare but devastating manifestation of MM.
